# Genetic control of flowering time in legumes

**DOI:** 10.3389/fpls.2015.00207

**Published:** 2015-04-09

**Authors:** James L. Weller, Raúl Ortega

**Affiliations:** School of Biological Sciences, University of Tasmania, Hobart, TASAustralia

**Keywords:** legume, flowering, photoperiod, pea, soybean

## Abstract

The timing of flowering, and in particular the degree to which it is responsive to the environment, is a key factor in the adaptation of a given species to various eco-geographic locations and agricultural practices. Flowering time variation has been documented in many crop legumes, and selection for specific variants has permitted significant expansion and improvement in cultivation, from prehistoric times to the present day. Recent advances in legume genomics have accelerated the process of gene identification and functional analysis, and opened up new prospects for a molecular understanding of flowering time adaptation in this important crop group. Within the legumes, two species have been prominent in flowering time studies; the vernalization-responsive long-day species pea (*Pisum sativum*) and the warm-season short-day plant soybean (*Glycine max*). Analysis of flowering in these species is now being complemented by reverse genetics capabilities in the model legumes *Medicago truncatula* and *Lotus japonicus*, and the emergence of genome-scale resources in a range of other legumes. This review will outline the insights gained from detailed forward genetic analysis of flowering time in pea and soybean, highlighting the importance of light perception, the circadian clock and the *FT* family of flowering integrators. It discusses the current state of knowledge on genetic mechanisms for photoperiod and vernalization response, and concludes with a broader discussion of flowering time adaptation across legumes generally.

## Introduction

Many aspects of plant growth and development are highly attuned to the environment, and this is particularly true of the transition from vegetative growth to the flowering state. Genetic variation that affects the timing of flowering and its regulation by environmental factors has clear significance for the performance of crop species. Many species have a requirement for exposure to specific photoperiods and/or temperatures in order to flower, and flowering may be significantly delayed or prevented if these requirements are not met. Genetic changes that relax or eliminate these constraints have enabled expansion to a wider latitudinal and climatic range and allowed greater flexibility in seasonal cropping practices. Conversely, delayed flowering may be an advantage in other situations, as it may reduce damage from certain abiotic stresses and lead to greater yield through increased biomass accumulation prior to flowering. Knowledge of how individual genetic variants combine to provide adaptation in specific situations can be valuable for breeding purposes, as it can accelerate introgression of new traits into adapted backgrounds and may allow the directed modification of phenology for specific target environments.

The major crop legumes fall within two sister clades that are included in the larger group of papilionoid legumes, often referred to as the galegoid and phaseoloid clades (Cronk et al., 2006). In general, species within the galegoid clade (e.g., pea, lentil, chickpea, faba bean) are from temperate regions and with respect to flowering time control are vernalization-responsive long-day plants (LDPs), whereas those in the phaseoloid clade (e.g., soybean, cowpea, pigeonpea, common bean) generally originate from lower latitudes and are short-day plants (SDPs; Summerfield and Roberts, 1985b). Two other important crop legumes, peanut (*Arachis hypogaea*) and lupin (*Lupinus* spp.) are basal to both of these clades. Not surprisingly, in view of their respective origins at low and high latitudes, peanut has characteristics of a SDP (Bagnall and King, 1991) and the commonly grown lupin species are vernalization-reponsive LDP (Summerfield and Roberts, 1985c).

While the defining feature of the vegetative-to-reproductive transition is the conversion of meristems to produce flowers rather than vegetative shoots, in a natural environment this is also accompanied by significant changes to a wide range of other developmental traits, including stem elongation, apical dominance, lateral branching, resource allocation, maturity and yield. In some species, genes and environmental factors that affect the initial flowering transition can also continue to have a significant influence on post-flowering processes affecting fertility and pod development. Thus, although flowering time can be seen from one perspective as a relatively simple trait, in reality it is linked to fundamental decisions made by the plant about when and how to allocate resources, and thus participates in a complex network of two-way interactions with other developmental processes.

Over recent years there has been a dramatic increase in the number of legume species with genome sequences and/or significant genomic resources (Young and Bharti, 2012). However, in the study of flowering time, as for other developmental processes, no one legume species has emerged as the predominant model. Most work on flowering time has focused on the two species pea (*Pisum sativum*) and soybean (*Glycine max*), which respectively represent the temperate LDP and warm-season SDP. This is partly due to the long history in the use of both of these species for flowering research and the accumulation of knowledge on its genetic control (Murfet, 1985; Summerfield and Roberts, 1985a; Weller et al., 1997b, 2009; Watanabe et al., 2012). Interestingly, the two legume model species *Medicago truncatula* (barrel medic) and *Lotus japonicus*, which have been prominent in studies of nitrogen fixation and other fundamental processes, and have genome sequences available, have so far not been deeply exploited for investigation of flowering time control.

## Flowering Genes

The availability of extensive genomic resources for several legume species and well-documented synteny has enabled a comprehensive inventory of genes potentially relevant for flowering time control. This has been useful both in identification of candidate genes for flowering loci, and in exploring the molecular physiology of flowering through gene expression studies and reverse genetics. It has also dramatically improved the prospects for discovering legume-specific genes through purely positional approaches. Well over 100 genes that contribute to control of flowering time have been identified in *Arabidopsis* and rice, and the functions and interactions of these genes are regularly reviewed (e.g., Kim et al., 2009; Amasino and Michaels, 2010; Andres and Coupland, 2012; Pin and Nilsson, 2012; Brambilla and Fornara, 2013; Song et al., 2013). In legumes, several compendia of flowering gene homologs are already available (Hecht et al., 2005; Kim et al., 2012; Watanabe et al., 2012). While the basic genes and gene families central to pathways controlling flowering time in *Arabidopsis* appear to be largely conserved in legumes, there are numerous examples of gene duplication and loss. This is likely to reflect the history and consequences of genome duplications after the divergence of the *Arabidopsis* and legume lineages (Young and Bharti, 2012).

Photoreceptors provide primary information about the light environment that enables detection of daylength. Legumes have a standard complement of only three phytochromes (phyA, phyB, and phyE; Hecht et al., 2005), and lack a representative of the ancient phyC clade, although the phyA lineage has undergone a more recent duplication in the phasioloid legumes (Liu et al., 2008). The cryptochrome gene CRY1 has also been duplicated in the phasioloid legumes, while an older duplication of the CRY2 gene is common to all legumes (Platten et al., 2005b). In contrast, only two LOV-domain flavoprotein photoreceptors in the FKF1/ZTL family are present in legumes. The circadian clock is also important for photoperiod measurement, and while all major *Arabidopsis* clock genes are represented, the galegoid legumes appear to have only a single gene orthologous to the circadian-clock related MYB transcription factor genes *CCA1* and *LHY* (Hecht et al., 2007), whereas the *TOC1*, *GI*, and *ELF3* genes have variously undergone duplication in the two legume groups. The CONSTANS protein, which integrates light and circadian signaling for photoperiod-specific induction in *Arabidopsis*, is represented by two co-orthologs in the SD legumes, but only one in the LD legumes (Wong et al., 2014).

Another feature of legumes is the expansion of the *FT*/*TFL1* gene family. In *Arabidopsis*, these genes integrate environmental signaling for induction of flowering, and guide the fate of meristems during inflorescence development. Both major crop legume groups have multiple *TFL1* genes and in the galegoid legumes two of these sequences are distinctively divergent from *Arabidopsis TFL1* (Foucher et al., 2003; Cronk et al., 2006; Hecht et al., 2011). Legumes also have three distinct subclades of *FT* genes; *FTa*, *FTb*, and *FTc* (Hecht et al., 2011). Within the large family of MADS domain genes, legumes have additional *SVP* and *SOC1* genes (Hecht et al., 2005; Jaudal et al., 2014), while the *FLC* clade, which has an important role in vernalization response in *Arabidopsis*, appears to be absent in the galegoid legumes and at most vestigial in soybean (Hecht et al., 2005; Ruelens et al., 2013).

## Genetic Analysis of Flowering in Legumes

### Pea (*Pisum sativum* L.)

Over 20 loci related to flowering time and inflorescence development have been identified in pea (**Table 1**). Initial work on genetic control of flowering resolved several loci from existing variation among various cultivars of garden and field pea, while other loci were subsequently identified through characterization of induced mutants and specific mutant screens (Murfet, 1985; Weller et al., 1997a, 2009).

**Table 1:** Comparison of flowering loci in pea and soybean.

Developmental role	Pea locus		Reference for molecular identity	Soybean locus		Reference for molecular identity	Molecular identity
Light perception/signaling	*FUN1*	+	Weller et al. (2004)	*E3*	-	Watanabe et al. (2009)	*PHYA*
				*E4*	-	Liu et al. (2008)	*PHYA*
	*LV*	-	Weller et al. (2001)				*PHYB*
	*LIP1*	-	Sullivan and Gray (2000)				*COP1*
Circadian clock	*SN*	-	Liew et al. (2014)				*LUX*
	*DNE*	-	Liew et al. (2009)				*ELF4*
	*PPD*	-					?
	*HR*	-	Weller et al. (2012)				*ELF3*
Photoperiod response	*E*	+					?
	*LATE1*	+	Hecht et al. (2007)	*E2*	-	Watanabe et al. (2011)	*GIGANTEA*
	*LATE2*	-					
				*E1*	-	Xia et al. (2012)	B3-domain TF
				*E7*	-		?
				*E8*	-		?
Signal integration and inflorescence development	*GIGAS*	+	Hecht et al. (2007)				*FT (FTa1)*
	*LF*	-	Foucher et al. (2003)				*TFL1 (TFL1c)*
	*VEG1*	+	Berbel et al. (2012)	*Dt2*		Ping et al. (2014)	MADS box TF
	*VEG2*	+	Sussmilch et al. (2015)				*FD*
	*LATE5*	+					
	*DET*		Foucher et al. (2003)	*Dt1*		Liu et al. (2010)	*TFL1 (TFL1a)*
	*UNI*		Hofer et al. (1997)				*LFY*
Other/unknown				*E5*	-		?
				*E6*	+		?
				*E9*	+		?
				*J*	+		?
	*LATE3*	+					?
	*LATE4*	+					?
	*LW*	+					?
	*AERO1*	-					?

*References establishing the molecular identity of each locus are cited. The effect (or inferred effect) on flowering of the functional (wild-type) allele is indicated as either “+” (promoting flowering) or “-” (inhibiting flowering). The molecular identity refers to the *Arabidopsis* ortholog or the type of protein encoded.*

#### Naturally Occurring Variation

Two major loci are known that delay flowering under non-inductive SD. Recessive alleles at the *HIGH RESPONSE (HR)* locus cause early flowering in SD and reduce, but do not eliminate, the photoperiod response, whereas recessive alleles at the *STERILE NODES (SN)* locus confer complete daylength insensitivity (Murfet, 1985). Only a single, naturally occurring mutant *hr* allele has been identified, but for *SN* both naturally occurring and induced mutant alleles have been described (Liew et al., 2014). Recent work has established *HR* and *SN* as pea orthologs of *Arabidopsis* circadian clock genes *ELF3* and *LUX*, respectively (Weller et al., 2012; Liew et al., 2014), and analysis of sequence diversity suggests a wide distribution of the *hr* allele across domesticated pea germplasm and an important and ancient role for this mutation in the spring-flowering habit (Weller et al., 2012). In contrast, the main naturally occurring *sn* allele has a more restricted distribution and occurs only within the subset of lines carrying *hr*, implying a more recent origin (Liew et al., 2014).

The third locus, *LATE FLOWERING* (*LF*), inhibits flowering in both long and short days, and was identified over 10 years ago as a divergent homolog of *TFL1* (Foucher et al., 2003). Numerous allelic variants of *LF* are known, including both naturally occurring and induced mutant alleles. Accessions in which the *LF* gene is deleted or inactivated by nonsense mutation show extremely early, photoperiod-insensitive initiation of flowering (Murfet, 1985; Foucher et al., 2003), but remain responsive to photoperiod in several other respects, suggesting that *LF* is not involved directly in the photoperiod response mechanism.

The fourth locus, *EARLY* (*E*), is the least well-understood of the naturally variant loci. Dominant alleles of *E* confer early initiation of flowering in some genetic backgrounds, but this effect shows complex interactions with other loci and incomplete penetrance. Recent identification of a major-effect quantitative trait loci (QTL) for flowering time in a chromosomal location similar to *E* (Lejeune-Hénaut et al., 2008; Weller et al., 2012) may help in its future molecular characterization.

Allelic differences at the *HR*, *SN*, *LF*, and *E* loci interact to specify an extremely wide range of flowering times in plants in non-inductive conditions. This range extends from the genotype *lf sn* which may flower as early as node 7 and is completely insensitive to photoperiod, to genotype *LF SN HR e* which flowers relatively late under LD and may not flower at all under SD (Murfet, 1985; Weller et al., 2012). Interestingly, most mutagenesis programs in pea have been conducted in spring-flowering (*hr*) cultivars, and in some cases in lines that also carry *sn* or *lf* alleles, and many are also likely to carry derived alleles at the *E* locus. Mutants isolated from these programs therefore carry at least one additional mutation affecting flowering time, and potentially as many as four.

#### Induced Mutants – Photoperiod Response

In addition to the *SN* and *HR* loci, several other photoperiod response loci have been identified through analysis of induced mutants. Two of these, *DIE NEUTRALIS* (*DNE*) and *PHOTOPERIOD* (*PPD*) have a role similar to *SN*, as recessive *dne* and *ppd* mutants show early flowering in SD. In the presence of *hr*, these mutations confer complete photoperiod insensitivity for flowering and other traits (King and Murfet, 1985; Arumingtyas and Murfet, 1994). *DNE* is the pea ortholog of another circadian clock gene, *ELF4* (Liew et al., 2009), but *PPD* has not yet been identified. In *Arabidopsis*, the proteins encoded by *ELF3*, *ELF4*, and *LUX* genes participate in the so-called evening loop of the circadian clock and work together in a complex termed the evening complex (EC; Nagel and Kay, 2012), which may provide a mechanistic explanation for the fact that mutants for *HR/PsELF3*, *DNE/PsELF4*, and *SN/PsLUX* have similar phenotypes.

Loci involved in promoting flowering in pea under inductive (LD) conditions have also been identified. Mutants for the phyA photoreceptor were first identified in screens for seedling photomorphogenesis and subsequently shown to have a LD-specific late-flowering phenotype (Weller et al., 1997a). The *phyA* mutants are largely insensitive to LD, although day extensions with artificial light rich in blue or far-red wavelengths can result in earlier flowering, implying a contribution from other photoreceptors (Weller et al., 1997a; Platten et al., 2005a). While phyB and cry1 might seem plausible candidates for this activity, evidence from *phyB* and *cry1* mutants suggests that neither is fundamentally involved in promotion of flowering (Weller et al., 2001; Platten et al., 2005a). The importance of *PHYA* is underlined by the dominant early flowering photoperiod-insensitive *phyA-3D* mutant, which has a higher level of phyA protein due to increased protein stability (Weller et al., 2004). Other loci *LATE1* and *LATE2* have been identified in specific mutant screens (Hecht et al., 2007). *LATE2* has not yet been identified, but *LATE1* is an ortholog of *Arabidopsis* circadian-clock-related gene *GIGANTEA (GI;* Hecht et al., 2007). The *late1* mutants are similar to *phyA* mutants with respect to their effect on flowering and photoperiod responsiveness, but have only mild photomorphogenic defects. Similar to *Arabidopsis GI*, *LATE1* shows strongly rhythmic expression, and *late1* mutants affect the expression rhythms of key circadian clock genes, confirming that *LATE1* has a role in clock function (Hecht et al., 2007; Kim et al., 2012; Weller et al., 2012; Liew et al., 2014).

Mutations at other pea loci affect the flowering transition without significantly interfering with the overall ability of the plant to respond to daylength. Under LD, *gigas* and *vegetative1* (*veg1*) and *veg2* mutants do not produce flowers, but instead show a profuse outgrowth of aerial vegetative branches (Murfet, 1985; Hecht et al., 2011; Berbel et al., 2012; Sussmilch et al., 2015). This phenotype appears to represent a failure to specify the identity of secondary inflorescences and to induce the expression of the floral meristem identity gene *PROLIFERATING INFLORESCENCE MERISTEM (PIM)*, a co-ortholog of *AP1* (Taylor et al., 2002; Berbel et al., 2012). *GIGAS* and *VEG1* have been respectively identified as an *FT* homolog and as a divergent member of the *AP1* clade of MADS-domain genes (Hecht et al., 2011; Berbel et al., 2012), and VEG2 was recently shown to be the pea ortholog of FD, an BZIP transcription factor that is an important signaling partner of FT proteins (Sussmilch et al., 2015).

### Soybean [*Glycine max* (L.) Merr.]

At least 10 loci that affect flowering-related characteristics in the SDP soybean have now been described (**Table 1**). Variation at these loci is responsible for a major proportion of the latitudinal adaptation in soybean, which is grown from tropical regions to 50°N. Cultivars grown at lower latitudes experience a longer growing season and are relatively late to mature, whereas expansion to higher latitudes and completion of the growth cycle within the short summer growing season have required a reduction in sensitivity to the inhibitory effects of LD.

The well-known *E* series of maturity loci (*E1* to *E9*) confer early flowering and maturity, particularly under non-inductive (LD) conditions (Cober and Morrison, 2010; Watanabe et al., 2012; Kong et al., 2014). With the exception of *E6* and the recently described *E9* locus, the early flowering alleles at the *E* loci are recessive (Watanabe et al., 2012; Kong et al., 2014). Addition of early flowering alleles at these loci results in incrementally earlier flowering under LD and improved adaptation to short summers at high latitudes. In contrast, “delayed juvenile” soybean lines are conspicuously later to flower under inductive SD conditions, a trait useful for adaptation to low latitudes generally and spring sowings at middle latitudes (Tomkins and Shipe, 1997). Genetic control of the delayed juvenile trait is not currently clear. One study proposed a single locus (*J*), with recessive alleles conferring late flowering in SD (Ray et al., 1995), whereas other analyses point to a more complex genetic control involving up to two other genes, one of which may be *E6* (Cober, 2011). Flowering time in soybean has also been analyzed as a quantitative trait, and several QTL have been identified, many of which are likely to correspond to known maturity loci (Watanabe et al., 2012). The *E1*, *E2*, and *E3* loci in particular appear to have been detected in a number of QTL studies and have also been referred to as *Flowering Time* 1 (*FT1*), *FT2*, and *FT3*, respectively (Yamanaka et al., 2000).

Several of the *E* loci have been characterized physiologically. As in pea, the effects of individual soybean loci have mostly been examined in genetic backgrounds already carrying hypomorphic alleles at other loci, making it difficult to gain a full picture of the action and interactions of any given locus. Nevertheless, five of the eight loci (*E1*, *E3*, *E4*, *E7, E8*) appear to specifically affect photoperiod responsiveness (Cober et al., 1996a, 2001, 2010; Cober and Voldeng, 2001). In particular, differential sensitivity of *E3* and *E4* loci to light quality of an artificial LD implicated them in the phytochrome system (Cober et al., 1996b), and both genes have subsequently been shown to encode phyA-type photoreceptors (Liu et al., 2008; Watanabe et al., 2009). Soybean contains four *PHYA* genes that consist of two pairs of homeologs, with *E3* and *E4* representing different homeolog pairs. The homeolog of *E4*, *PHYA1*, is apparently functional, whereas the homeolog of *E3* carries a deletion and is probably a pseudogene (Watanabe et al., 2009). In most plant systems, phyA is important for de-etiolation under continuous far-red (FR) light. Interestingly, loss of *E4* function reduced but did not abolish de-etiolation under FR, whereas loss of *E3* function had no effect on this response, even in the absence of *E4* (Liu et al., 2008). This suggests that the FR-sensing role during de-etiolation has become subfunctionalized to the *E4*/*PHYA1* pair, and implies that phyA1 may also contribute to photomorphogenic responses. The presence of phyA1 may also explain why the *e3 e4* mutant still shows delayed flowering in response to photoperiod extensions rich in far-red light. However, it is clear that three other loci, *E1*, *E7*, and *E8* also contribute to this response (Cober and Voldeng, 2001; Cober et al., 2010).

Like the pea *LATE1* locus, soybean *E2*/*FT2* was also identified as an ortholog of *GIGANTEA*, through a strategy involving fine mapping, candidate gene analysis and reverse genetics (Watanabe et al., 2011). Although recessive *e2* alleles can promote flowering under both LD and SD, *E2* is also reported to enhance the photoperiod response and clearly contributes to early flowering and latitudinal adaptation (Jiang et al., 2014). However, one of the most significant developments to emerge from analysis of soybean flowering loci has been the recent molecular characterization of the *E1* gene. *E1* has a major role in natural variation for flowering in soybean and has the largest effect among the *E* loci (Yamanaka et al., 2000). Positional cloning of *E1* revealed that it possesses a region of weak similarity to the plant-specific B3 domain, in addition to a helix-turn-helix domain and a nuclear localization signal, all suggesting a probable role as a transcription factor (Xia et al., 2012). *E1* essentially appears to define a legume-specific gene family, but it is distantly related to the RAV subfamily of B3 domain proteins, which includes the *Arabidopsis TEMPRANILLO* genes that are also transcriptional repressors of FT (Castillejo and Pelaz, 2008).

## Flowering Pathways in Legumes

### The FT Gene Family

*FT* genes are of particular interest for understanding flowering time control, in view of their well-documented roles in integration of environmental signals for flowering and in signaling from the site of photoperiod detection in the leaf to the site of flower formation at the shoot apex (Pin and Nilsson, 2012). The *FT* gene family has been studied in detail in pea, *Medicago*, *Lotus*, and soybean (Kong et al., 2010; Laurie et al., 2011; Yamashino et al., 2013; Zhai et al., 2014). Most species have at least five *FT*-like genes that comprise three distinct clades unique to legumes; *FTa*, *FTb*, and *FTc* (Hecht et al., 2011). Genes in the *FTc* group are the most divergent, and are distinguished from *Arabidopsis FT* and most other *FT* genes by substitution of several conserved residues.

**FIGURE 1:**
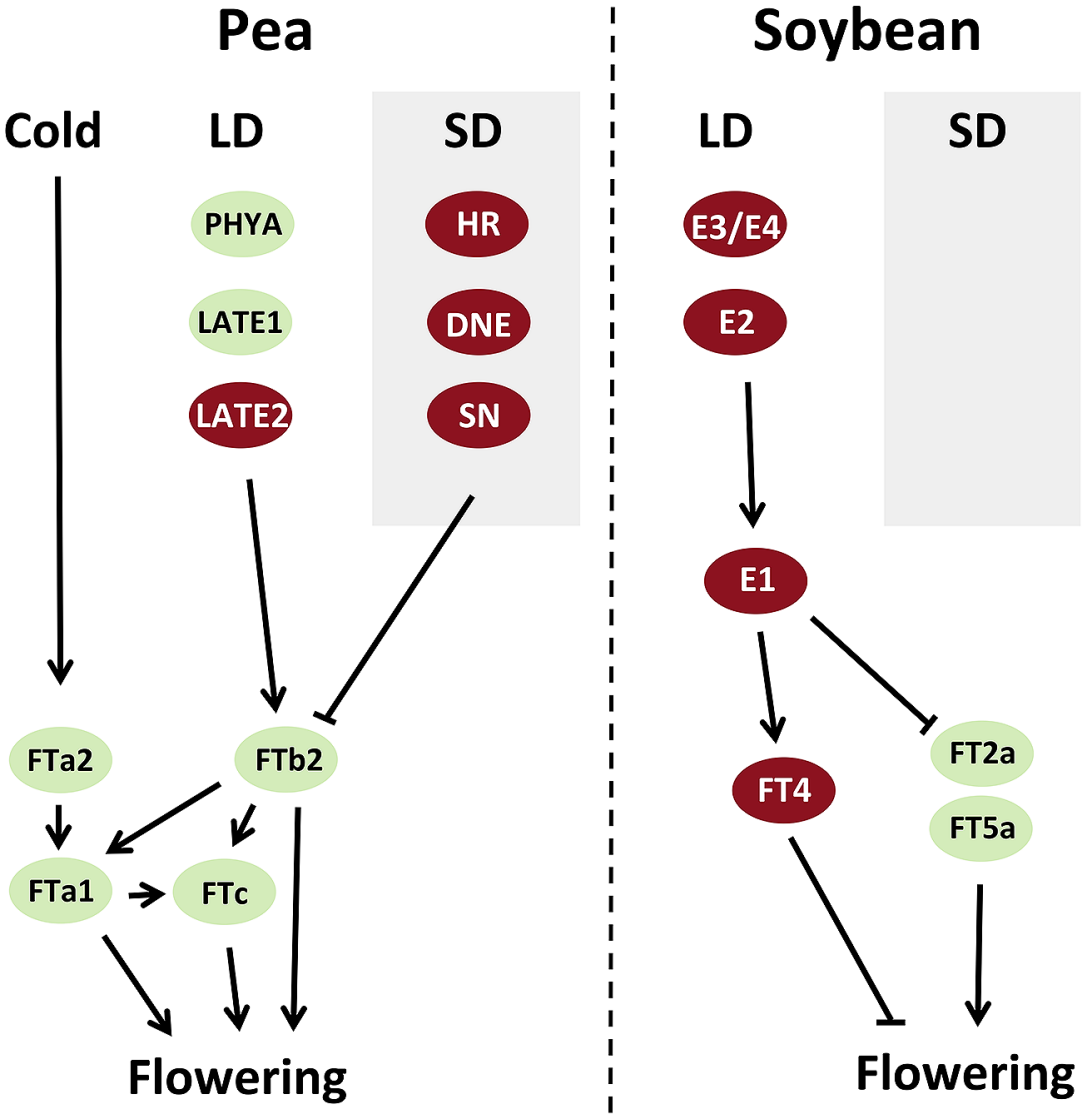
**Models summarizing interactions between flowering genes in control of flowering time in pea and soybean**. Genes that promote flowering are shown in green, and those that inhibit flowering are shown in red. LDs, long days; SDs, short days.

In pea and *Medicago*, study of expression patterns and mutant phenotypes suggest that *FTa* and *FTb* genes are expressed in leaves and may be important for targets of vernalization and photoperiod responses respectively, whereas *FTc* genes are expressed only in the shoot apex and may contribute to the integration signals from leaf-expressed *FT* genes (Hecht et al., 2011; Laurie et al., 2011). All pea *FT* genes can promote flowering to some extent when expressed in transgenic *Arabidopsis* (Hecht et al., 2011) but one in particular, *FTb2*, meets the characteristics of the classical “florigen.” It is expressed specifically in leaves under LD, and its upregulation correlates closely with the physiological induction of flowering and precedes the induction of all other *FT* genes. In addition, its expression correlates strongly with production of a graft-transmissible flowering stimulus (Hecht et al., 2011). Another pea *FT* gene, *FTa1*/*GIGAS*, is also expressed in leaves, and grafting experiments suggest that it may also generate a mobile flowering signal (Beveridge and Murfet, 1996; Hecht et al., 2011). However, the timing of *FTa1* induction is delayed relative to *FTb2*, and *gigas* mutants have inflorescence identity defects but respond strongly to daylength. This suggests that the two main *FT* genes expressed in leaves in pea may both signal to the shoot apex but have different developmental roles. In addition, it appears that pea *FT* genes may regulate each other (Hecht et al., 2011; **Figure 1**).

In soybean, two *FT* genes have been singled out as important promoters of flowering; *FT2a* (an *FTa* gene) and *FT5a* (an *FTc* gene). Expression of both genes is induced in leaves under inductive (SD) photoperiods, and both promote flowering when overexpressed in either *Arabidopsis* or soybean itself (Kong et al., 2010; Nan et al., 2014). Whereas *FT2a* appears to be qualitatively regulated by photoperiod, *FT5a* is expressed to some extent even in LD suggesting its role may not be restricted to photoperiod response (Kong et al., 2010). Two other *FTa* genes (*FT3a* and *FT3b*) also show significant expression in leaves in SD suggesting they may also participate in promotion of flowering (Kong et al., 2010). Surprisingly, one of the four soybean *FTb* genes, *FT4*, shows an opposite pattern of regulation and appears to act as a repressor of flowering. *FT4* is induced in leaves in LD, and is able to delay flowering when overexpressed in *Arabidopsis* (Zhai et al., 2014). Interestingly, FT4 carries a substitution of a highly conserved glycine (G133R) in an important functional region, and the significance of this is supported by the fact that the same residue is also substituted in another FT known to repress flowering; FT1 in sugar beet (Pin et al., 2010). However, within the legumes the existence of a repressive FT may be unique to soybean, as all other legume FTb-type sequences known to date carry the canonical glycine in this position.

### Response to Photoperiod

In species as diverse as *Arabidopsis* and rice, the photoperiod-dependent induction of *FT* genes relies on interactions between light perception and the circadian clock (Andres and Coupland, 2012; Brambilla and Fornara, 2013; Song et al., 2013). It is likely that this is also the case in legumes, in view of the fact that orthologs of *PHYA* (a photoreceptor) and *GI* (a gene affecting clock function) are important regulators of photoperiodic flowering in both pea and soybean (Weller et al., 1997a; Hecht et al., 2007; Liu et al., 2008; Watanabe et al., 2009, 2011). The importance of the clock for legume photoperiod responsiveness is further reinforced by the fact that the pea *HR*, *DNE*, and *SN* genes are all orthologs of clock genes and influence clock function (Liew et al., 2009, 2014; Weller et al., 2012). In both soybean and pea, functional variation in *PHYA* and *GI* orthologs is clearly associated with differences in the expression of *FT* genes. Expression of soybean *FT2a* is elevated by *e2*, *e3*, and *e4* alleles under long days (Kong et al., 2010; Watanabe et al., 2011) indicating that all three of these *E* loci participate in a pathway for photoperiod response that converges on *FT2a*. In the pea *late1* and *phyA* mutants, expression of *FTb2* is not detected and other *FT* genes are more weakly expressed (Hecht et al., 2011), whereas expression of multiple *FT* genes is elevated in *dne* and *sn* mutants (Liew et al., 2009, 2014). These interactions are summarized in **Figure 1**.

Unfortunately, we do not yet have a clear picture of how these clock and photoreceptor inputs are integrated to provide photoperiod-specific regulation of *FT* genes. The paradigm for integration of light and clock signals in photoperiod measurement has been established in *Arabidopsis* and centers on the B-box transcription factor CONSTANS (CO), which is a direct transcriptional activator of *FT* (Tiwari et al., 2010). LD-specific induction of *FT* is achieved through transcriptional and post-translational regulation of CO. GI associates with the blue-light photoreceptor FKF1 to provide light-dependent promotion of *CO* transcription via degradation of the CDF family of CO transcriptional repressors (Andres and Coupland, 2012; Song et al., 2013). In contrast, phyA acts at the post-translational level by stabilizing CO protein under FR light, possibly through antagonism of CO degradation by the COP1 ubiquitin ligase complex (Andres and Coupland, 2012; Song et al., 2013) Although the GI-CO-FT regulatory relationship is also functionally significant in the SDP rice (Brambilla and Fornara, 2013), evidence for the more general conservation of this mechanism across flowering plants is limited (Ballerini and Kramer, 2011), which raises the question of whether it may operate in legumes.

Soybean contains four genes orthologous to *Arabidopsis CO* (Wong et al., 2014) and all four are reported to promote flowering in transgenic *Arabidopsis* (Wu et al., 2014), but their endogenous role has not yet been determined. In addition, although the *GmCOL1a* and *GmCOL1b* genes showed higher expression levels under SD (Wu et al., 2014) and show some overlap with *FT* genes in their diurnal expression pattern, it is still unclear whether these genes are transcriptionally regulated by any of the *E* loci or whether they in turn regulate any of the *FT* genes. The situation in the galegoid legumes is simpler, with only a single *CO* ortholog (*COLa*) known to be present (Hecht et al., 2005; Yamashino et al., 2013; Wong et al., 2014). While the function of this gene has not been directly investigated in pea, its expression is not misregulated in either *late1* (*Psgi*) or *dne* (*Pself4*) mutants, despite strong defects in *FT* regulation, flowering and photoperiod responsiveness in both mutants (Hecht et al., 2007, 2011; Liew et al., 2009). In addition, expression analyses, transgenic studies and characterization of specific mutants in *M. truncatula* also indicate that *COLa* does not have any substantial role in flowering time regulation, and suggest that the same is probably true for other *COL* genes (Wong et al., 2014).

The available evidence so far thus indicates that legume *CO*-like genes may not have a major role in integration of responses to photoperiod in the temperate legume group, and suggests that some alternative mechanism must be operating. One possibility is that the *E1* gene may participate in this role. Analysis of soybean *e1* mutants and *E1* overexpression lines clearly show that *E1* regulates expression of *FT* genes, including repression of *FT2a* and *FT5a* (Thakare et al., 2011; Xia et al., 2012) and induction of the repressive *FT4* (Zhai et al., 2014; **Figure 1**). *E1* is specifically expressed under LD, where it shows a strong diurnal expression rhythm, and is also transcriptionally regulated by *E4* (Xia et al., 2012), suggesting that regulation by light and the circadian clock may be important for its function. In future, it will be interesting to learn whether *E1* is also regulated by *E2* and *E3*, and whether *E1* orthologs also regulate flowering in the temperate legumes. However, even if *E1* does participate as a key integrator in the soybean photoperiod response mechanism, the fact that it is a repressor of *FT* suggests that there still may be undiscovered components required for upregulation of *FT* genes in inductive conditions.

One possible scenario for CO-independent FT induction in legumes is provided by a recent study in *Arabidopsis* which showed that GI can bind directly to the *FT* promoter to activate *FT* transcription in a CO-independent manner (Sawa and Kay, 2011), and it can also interact physically with TEM proteins, which are direct transcriptional repressors of *FT* distantly related to E1 (Castillejo and Pelaz, 2008; Sawa and Kay, 2011). It is possible that legume E1 and GI orthologs might interact in a similar way. Another scenario may be that the DNA binding and protein-interacting properties of CO may be partitioned into two separate proteins, as has recently been suggested in sugarbeet, where a CCT-domain pseudo-response regulator (PRR) protein (similar to the C-terminal domain of CO) and a B-box zinc finger protein (similar to the N-terminal domain of CO) may interact to confer CO function (Dally et al., 2014).

In addition, the fact that *PHYA* and *GI* orthologs are central components of the photoperiod response mechanism in both LDP pea and SDP soybean, but show opposite effects on flowering and *FT* expression in these two species, implies that the SD/LD difference results from a reversed regulatory interaction at some point downstream of both genes. The relatively close taxonomic relationship between these two species make them an attractive model for understanding the evolution of this difference in photoperiod response mode.

### Response to Vernalization

Regulation of flowering by vernalization is a phenomenon widespread across annual species from temperate regions, but is thought to have evolved independently in different plant lineages. As a result, the genes and genetic mechanisms conferring vernalization responsiveness are likely to differ across different groups (Kim et al., 2009). In legumes, the first insight into the genetic control of vernalization response has come from work in *M. truncatula*, where a survey of *Medicago FT* genes showed that two tandemly arranged *FT* genes (*FTa1* and *FTa2*) are induced by vernalization but have different temporal patterns of response (Laurie et al., 2011). *FTa2* is induced during exposure to cold, but *FTa1* is only expressed following return to warm conditions. Loss-of-function *FTa1* insertion mutants are insensitive to vernalization but retain sensitivity to photoperiod, indicating that *FTa1* is necessary for response to vernalization, and may be the key target in the legume vernalization pathway (**Figure 1**; Laurie et al., 2011). Interestingly, lines carrying insertions close to but not within *FTa1* coding regions show dominant inheritance of early, vernalization-independent flowering, which suggests that *FTa1* is normally subject to repression by adjacent regulatory regions and that this repression is normally overcome by vernalization (Jaudal et al., 2013).

## Control of Flowering in Other Legumes

Flowering time control is a key issue in adaptation of many other crop legumes (Summerfield and Roberts, 1985b; Nelson et al., 2010), and loci controlling flowering time and other flowering-related traits have now been identified in most crop and model legumes (**Table 2**). The majority of these loci have been detected as QTL, but in some cases they have been amenable to classical genetic analyses and Mendelian inheritance has been defined. It is likely that some of the same loci have been detected in different studies but it is difficult to assess this due to the lack of markers in common and/or sequence information.

**Table 2:** List of flowering time QTL in legumes, indicating the linkage group (LG) on which they occur.

Crop	LG	Reference
**Chickpea**
	1	Lichtenzveig et al. (2006) (1A), Rehman et al. (2011), Varshney et al. (2014) (1B)
	2	Lichtenzveig et al. (2006) (2A)
	3	Cobos et al. (2009) (3A), Aryamanesh et al. (2010) (3A, 3B), Hossain et al. (2010) (3C), Rehman et al. (2011) (3D), Varshney et al. (2014)
	4	Cobos et al. (2007), Rehman et al. (2011)
	(5)	Jamalabadi et al. (2013)
	8	Rehman et al. (2011) (8A), Varshney et al. (2014) (8B)
	?	Cho et al. (2002), Lichtenzveig et al. (2006), Vadez et al. (2012)
**Faba bean**
	1	Cruz-Izquierdo et al. (2012)
	5	Cruz-Izquierdo et al. (2012)
***Lotus***
	1	Gondo et al. (2007)
**Lupin**
	10	Kroc et al. (2014)
**Barrel medic**
	1	Pierre et al. (2008)
	4	Pierre et al. (2008)
	5	Pierre et al. (2008)
	7	Pierre et al. (2008)
	8	Pierre et al. (2008)
**Common bean**
	1	Blair et al. (2006), Chavarro and Blair (2010), Pérez-Vega et al. (2010)
	2	Blair et al. (2006), Pérez-Vega et al. (2010), Chavarro and Blair (2010)
	3	Chavarro and Blair (2010)
	6	Blair et al. (2006)
	7	Chavarro and Blair (2010)
	8	Pérez-Vega et al. (2010)
	9	Tar’an et al. (2002), Blair et al. (2006)
	11	Blair et al. (2006)
**Cowpea**
	2	Kongjaimun et al. (2012), Andargie et al. (2013, 2014)
	4	Xu et al. (2013)
	7	Andargie et al. (2013, 2014)
	10	Kongjaimun et al. (2012), Andargie et al. (2013), Xu et al. (2013)
	11	Kongjaimun et al. (2012), Xu et al. (2013)
**Azuki bean**
	2	Kaga et al. (2008)
	3	Kaga et al. (2008)
	4	Isemura et al. (2007), Kaga et al. (2008)
	5	Kaga et al. (2008)
	11	Kaga et al. (2008)
**Mungbean**
	2	Isemura et al. (2012), Kajonphol et al. (2012)
	4	Isemura et al. (2012), Kajonphol et al. (2012)
	6	Isemura et al. (2012)
**Pigeon pea**
	4	Kumawat et al. (2012)
	5	Kumawat et al. (2012)

**FIGURE 2:**
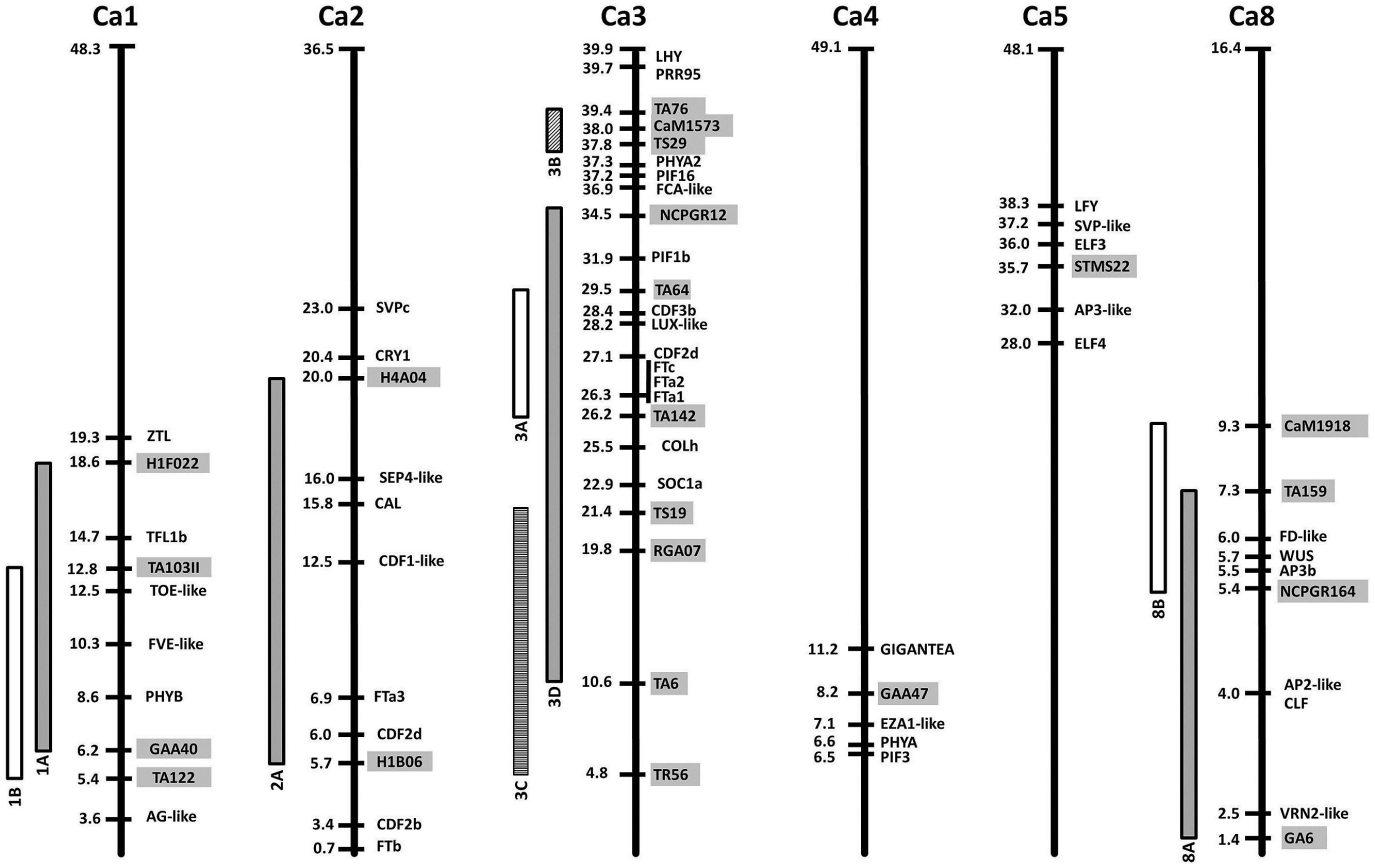
**Relative locations of flowering time gene homologs and quantitative trait loci (QTL) in the chickpea genome**. Chromosomes are represented as bold vertical lines with marker/gene positions indicated in Mbp. QTL are shown as vertical bars, and the markers delimiting them are indicated by gray boxes. References for each QTL can be found in **Table 2**.

*For chickpea, QTL on the same LG identified in different studies are distinguished by the LG number followed by a letter. More detail on these QTL is shown in **Figure 2**.*

While all of this variation is naturally arising and therefore likely to be adaptive, several loci can be singled out for a particularly significant contribution to adaptation and range expansion. One example is the SDP common bean (*Phaseolus vulgaris*), where, like soybean, expansion to higher latitudes has been accompanied by earlier flowering under LD and a reduction in photoperiod responsiveness (Gepts and Debouck, 1991). A significant proportion of this variation can be attributed to *PPD*, a mendelized locus on LG1, where recessive alleles confer reduced photoperiod response and early flowering under LD (Koinange et al., 1996). A second example is narrow-leafed lupin (*Lupinus angustifolius*), where the acquisition of early, vernalization insensitive flowering conditioned by dominant alleles at the the *Ku* locus has been integral to deployment of this crop for Mediterranean climates with mild winters in which a vernalization requirement would not be met (Nelson et al., 2010). A third example is lentil (*Lens culinaris*), where an early flowering variant at the *Sn* locus has had an important role in developing early flowering cultivars for water-limited environments and broadening the genetic base of lentil in south Asia (Sarker and Erskine, 2006).

There is significant potential for translation of insights from the pea and soybean systems to achieve a better understanding of other legume species. This potential reflects advances in two areas. First, functional and phylogenetic analyses of flowering genes and gene families in pea and soybean can improve the identification of plausible candidate genes for particular loci. Second, availability of sequenced genomes and gene-based genetic maps have improved the technical ability to identify and evaluate candidate genes under QTL, and to identify those QTL that may have conserved locations across several species. In addition the fact that certain desirable traits such as early, photoperiod-insensitive flowering or determinate growth can result from simple monogenic loss-of-function mutations means that in species where such variants do not already exist it may be feasible to generate them through mutagenesis.

Within the legumes the value of a comparative approach is most clearly shown by recent findings that in several species, determinate inflorescence architecture is conferred by mutation of specific TFL1 genes [described by Benlloch et al. (2015) in this Research Topic]. A second example is the recent identification of the lentil *SN* locus as the ortholog of pea *HR* (Weller et al., 2012), suggesting that the nature of flowering time adaptation may be widely conserved. In addition to these well-established examples, it is now also possible to identify other potentially conserved QTL and to begin to speculate about their nature (**Figure 2**).

The most prominent case is the existence of a conserved major flowering time QTL in a region syntenic with a section of *Medicago* chromosome 7 containing a tandem array of *FTa* and *FTc* genes. This region is now implicated in control of flowering time in numerous members of the temperate legume clade, including *M. truncatula* itself (Pierre et al., 2008), faba bean (Cruz-Izquierdo et al., 2012), chickpea (Cobos et al., 2009; Aryamanesh et al., 2010), narrow-leafed lupin (Nelson et al., 2006), *L. japonicus* (Gondo et al., 2007) and alfalfa (Robins et al., 2007). As described above, functional studies in *Medicago* and pea have demonstrated the importance of *FT* genes in this cluster for vernalization responsiveness and other aspects of flowering (Laurie et al., 2011), and this implies that disruption to one or more of these genes is the most likely molecular basis for these QTL. Evidence from *Medicago* and pea indicates that *FTa* and *FTc* genes promote flowering, and a simple loss-of-function mutation would therefore be expected to be recessive and late-flowering. However, for several of these QTL the “derived” form appears to be early flowering, as the later-flowering variant is much more widespread and likely to be ancestral (faba bean, chickpea, lupin), and at least in lupin and chickpea the early flowering trait has been reported to show dominant inheritance (Aryamanesh et al., 2010; Nelson et al., 2010). These observations suggest that if genes in the *FTa/c* cluster are the basis for these QTL, the genetic changes are likely to be gain-of-function mutations, and it might also be expected that one or more of the *FT* genes in the cluster would show high and/or de-regulated expression. This also suggests that the causal genetic changes could be varied and complex, potentially involving variation in copy number or alteration to promoter or other regulatory regions, as observed for gain-of-function mutations affecting flowering time genes in cereals (e.g., Beales et al., 2007; Nitcher et al., 2013).

A closer look at individual species provides additional illustrations of a comparative approach to candidate gene identification. The first example is common bean where, in addition to several flowering time QTL (**Table 2**), two loci controlling photoperiod response have also been characterized; *Ppd* and *Hr* (Gu et al., 1998). The *Ppd* locus has been mapped to within 5 cM of the *FIN* locus controlling shoot determinacy (Kwak et al., 2008), and the molecular identity of *FIN* as a *TFL1* co-ortholog (Repinski et al., 2012) therefore indicates the approximate genomic region in which *PPD* is located. This region is syntenic with the region in soybean containing the *E3/PHYA3* gene (McClean et al., 2010) and, as expected, contains the bean *E3* ortholog, suggesting this as an attractive candidate for the *PPD* locus. A second bean locus contributing to photoperiod sensitivity, *Hr*, is less well-defined but is positioned toward the other end of the same linkage group (Gu et al., 1998), a region that contains homologs of *ELF3* and the *FTa*/*c* cluster. A third locus identified as a QTL in LG9 is located near the bean ortholog of *ZEITLUPE*, a gene important for circadian clock regulation in *Arabidopsis* (Tar’an et al., 2002; Kwak et al., 2008).

The second example is chickpea, where classical genetic analyses have distinguished four Mendelian loci, named *Early flowering* 1 (*Efl1*) to *Efl4* (Gaur et al., 2015). Recessive alleles at these loci confer early flowering and at least two are likely to be widespread within the chickpea germplasm and have a major impact on flowering time adaptation. In addition, many linkage studies have been performed in chickpea, and six flowering time QTL have been defined in LG1, 2, 3, 4, and 8 (**Table 2**). Some of these studies have involved parents known to carry *efl1* or *efl2*, suggesting that QTL may correspond to major loci in some cases. This, together with the availability of the chickpea genome sequence (Jain et al., 2013; Varshney et al., 2013) enables an assessment of approximate co-location between flowering gene homologs and flowering time loci or QTL (**Figure 2**). For example, *Efl1* was defined in a cross using the early flowering line ICCV2 (Kumar and van Rheenen, 2000), which has also been used as a common parent in several studies reporting a major QTL (Cho et al., 2002; Vadez et al., 2012; Jamalabadi et al., 2013). Although its genomic location is still uncertain (as markers near the QTL have been variously assigned to LG3, 4, 5, 6, and 8), the clearest and most recent indication is given by Jamalabadi et al. (2013) who placed a major QTL between markers TA117 and STMS22, which both map to LG5 (Radhika et al., 2007; Hiremath et al., 2012). The latter is physically located near genes responsible for flowering time variation in other species such as *ELF3* or *ELF4*, but also *LEAFY* (*LFY*), which is involved in flower initiation and inflorescence development (Hofer et al., 1997). Recessive alleles at the photoperiod response locus *Efl2* are present in the cultivar ICC5810 (Or et al., 1999), a line also used as a parent by Lichtenzveig et al. (2006), who found a major QTL in LG1 explaining 60% of the flowering time variation. This chromosomal region contains several genes with the potential to explain these differences, including orthologs of *PHYB* and *TFL1*.

For the remaining reported QTL, there is no clear relationship to major flowering loci, but some potential candidates can be identified, including *PHYA* and *GI* for the LG4 QTL, another member of the *FT* family (*FTa3*) for the LG2 QTL, and a *FD*-like gene for the LG8 QTL (**Figure 2**). The QTL in the central region of LG3 deserves special attention as it has been reported several times (**Table 2**). The presence of the *FTa/c* cluster as the most likely candidate has been already mentioned, but other genes within the interval are also noteworthy, including, two *CYCLING DOF FACTOR* (*CDF*) homologs, a *LUX*-like gene, and an ortholog of the *CO*-like gene *COLh*. This last gene has also been highlighted as a candidate near the chromosome 7 QTL in *M. truncatula* (Pierre et al., 2011). A second QTL close to the bottom of chickpea LG3 has also been described (Aryamanesh et al., 2010), in a region containing genes involved in circadian clock and light signaling, including PRR and phytochrome-interacting factor (PIF) homologs, a *PHYA* paralog, and the ortholog of *LATE ELONGATED HYPOCOTYL* (*LHY*).

## Conclusion

Detailed genetic studies in model legumes are bringing rapid progress to our understanding of flowering time control, and have identified significant similarities and differences with other plant groups. At the same time, new genome sequences and dense, gene-based genetic maps are accelerating the translation of these insights to a range of other legume species, and giving new insight into how flowering time gene functions may be conserved or diversified within this important group of crops. It now seems reasonable to expect that genes underlying most of the major flowering time loci in legumes will be identified within the next 5–10 years, which will help improve our understanding of how they work together to provide adaptation to specific locations and agronomic constraints.

## Conflict of Interest Statement

The authors declare that the research was conducted in the absence of any commercial or financial relationships that could be construed as a potential conflict of interest.
